# Vaccine-induced pseudothrombocytopenia after Ad26.COV2.S vaccination

**DOI:** 10.1007/s00277-021-04611-y

**Published:** 2021-08-10

**Authors:** Marcel Kemper, Christoph Berssenbrügge, Georg Lenz, Rolf Michael Mesters

**Affiliations:** 1 Department of Medicine A, Hematology, Oncology, Hemostaseology and Pneumology, University Hospital Muenster, Albert-Schweitzer-Campus 1, 48149 Muenster, Germany; 2 General Practitioner, Bittgang 28, 49393 Lohne, Germany

Dear Editor,

Many cases of vaccine-induced immune thrombotic thrombocytopenia (VITT) have been reported after vaccination with the adenoviral vector vaccine ChAdOx1 nCov-19 (AstraZeneca) against severe acute respiratory syndrome coronavirus 2 (SARS-CoV-2) [1–3]. Recently, similar cases of thrombotic thrombocytopenia have been reported in patients after vaccination with the recombinant adenoviral vector vaccine Ad26.COV2.S (Johnson & Johnson/Janssen) [4, 5].

**Fig. 1:**
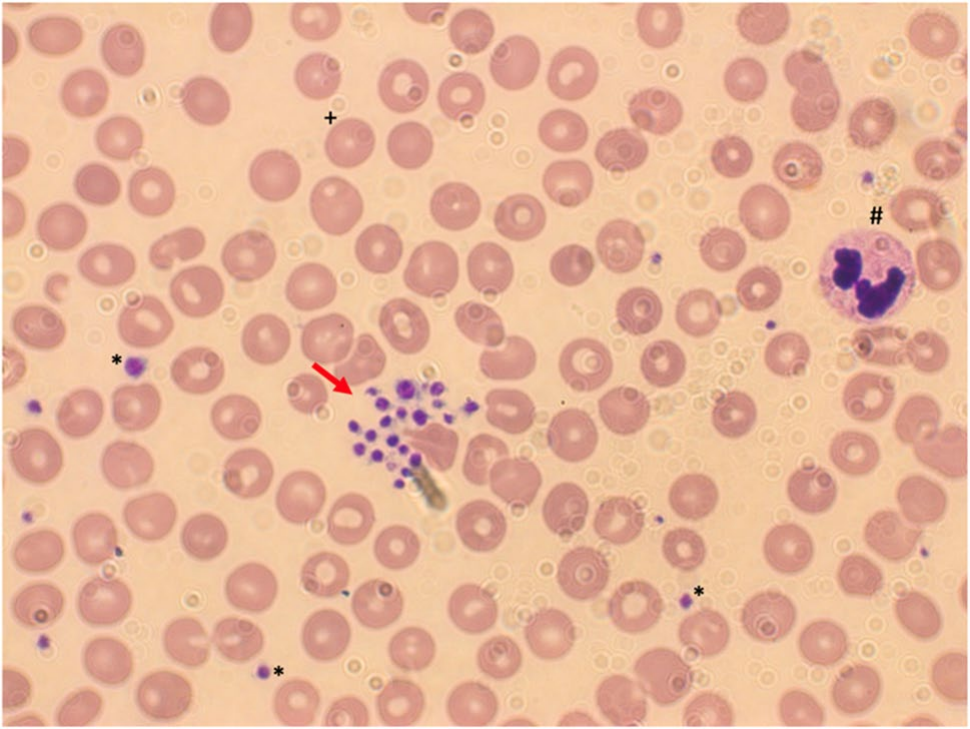
Peripheral blood smear from the EDTA-anticoagulated patient´s blood showing platelet aggregates (arrow), single platelets (*), red blood cells ( +), and a neutrophile (#) (100 × magnification)

A 38-year-old female patient presented at her family doctor on May 5th, 2021 with two small hematomas on her thighs after mild trauma and a red spot at the belly, which later on revealed as a telangiectasia. She also mentioned gingiva bleeding, which already occurred occasionally for many years. On May 1st, 2021, the patient was vaccinated with the adenoviral vector vaccine Ad26.COV2.S against SARS-CoV-2. A blood count showed mild thrombocytopenia (113.000/µl). On the next day, the platelet count further dropped to 15.000/µl, and the patient was immediately transferred to the emergency department of our University Hospital due to suspected VITT. We confirmed low platelet count (35.000/µl) in an EDTA (ethylenediaminetetraacetic acid) tube. D-dimer levels and further coagulation parameters ranged normal. Clinical examination revealed no signs for cerebral vein thrombosis. Deep vein thrombosis was excluded by venous ultrasound. To exclude pseudothrombocytopenia, we performed platelet count with the S-Monovette® ThromboExact (Sarstedt). Surprisingly, the platelet count revealed normal (210.000/µl), suspecting pseudothrombocytopenia in the patient. Platelet aggregates were confirmed by a blood smear (Fig. 1). Investigation of antibodies against the PF4-heparin/complex using the Asserachrom® HPIA-IgG (Stago) enzyme-linked immunosorbent assay (ELISA) resulted negative, excluding VITT. As the patient was suffering from psoriatic arthritis which was treated with methotrexate (MTX) 15 mg weekly, blood counts were taken regularly every 3 months, showing normal platelet counts in EDTA tubes before vaccination. Follow-up blood counts confirmed transient vaccine-induced pseudothrombocytopenia (VIP), as platelet counts in EDTA-anticoagulated blood normalized rapidly after a couple of days (201.000/µl on May 18th and 192.000/µl on June 25th).

In conclusion, here we report the first case of a 38-year-old female patient, who developed transient vaccine-induced pseudothrombocytopenia (VIP) after vaccination with Ad26.COV2.S. Due to the risk of overtreatment when pseudothrombocytopenia is not detected, we strongly recommend to exclude pseudothrombocytopenia in all patients with thrombocytopenia after vaccination with Ad26.COV2.S or ChAdOx1 nCov-19, especially before application of high-dose intravenous immunoglobulins and implementation of therapeutic anticoagulation. VIP seems to be a rare differential diagnosis to VITT.
